# To swim or not to swim: A population-level model of Xenopus tadpole decision making and locomotor behaviour

**DOI:** 10.1016/j.biosystems.2017.07.004

**Published:** 2017-11

**Authors:** Roman Borisyuk, Robert Merrison-Hort, Steve R. Soffe, Stella Koutsikou, Wen-Chang Li

**Affiliations:** a School of Computing, Electronics and Mathematics, University of Plymouth, Drake Circus, Plymouth, PL4 8AA, UK; b School of Biological Sciences, 24 Tyndall Avenue, University of Bristol, Bristol, BS8 1TQ, UK,; c School of Psychology & Neuroscience, Westburn Lane, University of St Andrews, St Andrews KY16 9JP, UK; d Institute of Mathematical Problems of Biology, The Branch of Keldysh Institute of Applied Mathematics of Russian Academy of Sciences, Pushchino, 142290, Russia

**Keywords:** Wilson-Cowan model, Sensory pathways, Central pattern generator, Swimming behaviour, Struggling behaviour

## Abstract

We present a detailed computational model of interacting neuronal populations that mimic the hatchling *Xenopus* tadpole nervous system. The model includes four sensory pathways, integrators of sensory information, and a central pattern generator (CPG) network. Sensory pathways of different modalities receive inputs from an “environment”; these inputs are then processed and integrated to select the most appropriate locomotor action. The CPG populations execute the selected action, generating output in motor neuron populations. Thus, the model describes a detailed and biologically plausible chain of information processing from external signals to sensors, sensory pathways, integration and decision-making, action selection and execution and finally, generation of appropriate motor activity and behaviour. We show how the model produces appropriate behaviours in response to a selected scenario, which consists of a sequence of “environmental” signals. These behaviours might be relatively complex due to noisy sensory pathways and the possibility of spontaneous actions.

## 1 Introduction

The hatchling *Xenopus* tadpole provides a good place to study decision making and behaviour because at this stage of development (approximately 2 days old, stage 37/38) the nervous system is relatively small (several thousand neurons), the behaviour is simple, and many biological details are known from experimental work.

In this paper, we present a new computational model of the tadpole nervous system that is informed by experimental data and is able to accurately reproduce tadpoles’ behaviour in response to input from multiple sensory modalities. We implement the integration of noisy sensory signals in a simple model of interconnected neuronal populations. This model can describe the behavioural switching observed in hatchling *Xenopus* tadpoles (Roberts et al., 2010). The aim of the model is to clarify the key universal neurobiological mechanisms and theoretical principles that underlie the decision-making process, as well as provide new ideas and hypotheses for experimental testing.

Many prevailing theories regarding decision making postulate that evidence from different sensory modalities is integrated until a threshold is reached and one of several actions is selected (Kristan, 2008, Marshall et al., 2012). This integration process is important due to the noisy nature of sensory signals. Our computational model describes the dynamics of behavioural responses to input signals from the “environment”. The modelling is based on multiple anatomical and neurophysiological findings regarding initiation of locomotion by trunk skin (Roberts et al., 2014) and head skin (Buhl et al., 2015) stimulation, as well as several other sensory inputs that are known to control tadpole locomotion (Roberts et al., 2010). The model includes two parts: (1) four sensory pathways (touch trunk skin, touch head, light dimming, and press head) and (2) the central pattern generator (CPG) neurons for execution of locomotor actions.

All sensory pathways in the model are organised in a similar way. A neuronal population corresponding to a particular sensory modality processes information from (non-modelled) sensory cells and delivers the result of this processing to a central integrator population. This integrator population is where decision-making and action selection occurs. The CPG populations work under the control of inputs from the integrated sensory signals to generate neuronal activity corresponding to one action from a repertoire including swimming, struggling, and accelerated swimming (Roberts et al., 2010; Li et al., 2015). The output of the CPG is motor neuron spiking, with different patterns of spikes corresponding to different actions.

Many experimental facts are known about hatchling *Xenopus* tadpoles regarding the different neuronal types that are present, including their anatomy, electrophysiology and synaptic connections. However, the amount of experimental data available is still not enough to produce a detailed single neuron level model of all sensory modalities and behaviours. Therefore, our approach is based on a mean-field (mesoscopic) model of neuronal activity.

The model is formulated as a system of 26 ordinary differential equations, which describe the average activity level in various interacting neuronal populations. The model is symmetrical, with 13 populations on the left-hand side of the body and 13 populations on the right-hand side. We describe the dynamics of population activity using the Wilson-Cowan model (Wilson and Cowan, 1972, Borisyuk and Kirillov, 1992). For each population we describe the dynamics of the average neuronal activity in the population of neurons in response to incoming synaptic inputs from the other populations. We use bifurcation analysis to determine the region in parameter space that corresponds to physiological activity such that the model produces the correct output. For example, for a pair of coupled populations, a region of parameter space where regular oscillations exist can be determined (Borisyuk and Kirillov, 1992). Bifurcation analysis also reveals how parameters can be changed to control the dynamics and switch from one dynamical mode to another.

Simulations show a good agreement between modelling results and experimental measures. For modelling swimming we use a bi-stable regime that exists in the system: a short-term input corresponding to stimulation of the trunk skin on one body side moves the system from a stable equilibrium to generating anti-phase oscillations (alternating left and right activity). This swimming mode exists for some time (if there are no perturbations from the sensory pathway) before spontaneously stopping and returning the system to the resting equilibrium. We show that the head touch sensory pathway can initiate swimming in a similar way.

According to experimental data, struggling behaviour is slower, stronger series of rhythmic trunk flexions seen while a tadpole is grasped by a predator (Roberts et al., 2010) and the corresponding spiking activity is of bursting type. To model struggling we use a dynamical regime of anti-phase envelope oscillations where the slow frequency relates to slow body movement and the fast frequency reflects bursting spiking. We show that this struggling mode in the model appears and exists during prolonged input from the skin touch sensory pathway.

Tadpoles have a pineal eye that is able to sense light. When the light sensed by this eye is dimmed, the swimming frequency increases (Jamieson and Roberts, 2000) which has the effect of causing the tadpole to swim upwards. The model mimics these experimental findings, increasing the activity rate of swimming transiently in response to input from the light dimming sensory pathway.

Experiments with the cement gland sensory pathway reveal that swimming stops when the cement gland is pressed or its mucus pulled (Roberts et al., 2010). A tadpole also stops swimming when it bumps into solid objects like vegetation or the side of a dish. Model simulations show that inhibitory input from the “head press” (cement gland) sensory pathway on one body side stops swimming and returns the system to the resting equilibrium.

We demonstrate that for a selected sequence of events (touch skin, dimming light, predator attack, head bump) reflecting a “natural” scenario (Li et al., 2014), the model correctly reproduces real CPG activity. These neuronal activities correspond to the tadpole’s behaviour. Thus, the model can generate proper behaviour in response to external events through the integration of sensory inputs and decision-making.

### 1.1 Repertoire of tadpole behaviours

In this section, we provide a short introduction to the set of possible tadpole behaviours (Li et al., 2014), which are reflected in our modelling.

In the resting state a tadpole is usually attached to some object (water surface, wall or bottom of a dish, etc). After about 20 s in this state the tadpole will drift to the bottom of the water and stay there for some time (about 60–90 s), and after that spontaneously start to swim (Jamieson and Roberts, 2000). Swimming consists of alternating left-right motor neuron activity at 10–25 Hz. If a tadpole is not moving (steady state) then swimming can start by one of the following ways: (1) on trunk skin touch; (2) on head skin touch; (3) on light dimming; (4) spontaneously. Swimming can stop: (1) on press head or (2) spontaneously.

Struggling behaviour is slower, stronger series of rhythmic trunk flexions seen while a predator grasps a tadpole. During struggling, active neurons fire bursts of spikes (Li et al., 2007). Each burst is about 100–200 ms long and the intra-burst spiking frequency is up to 245 Hz. Struggling can start (Soffe, 1991) in response to prolonged stimulation of trunk/head skin on both sides simultaneously, either when the tadpole is at rest or swimming. Struggling lasts during the stimulation period only and after that the tadpole switches to swimming.

When a tadpole is swimming, dimming the light causes the frequency of swimming to increase, which causes the animal to swim upwards (Jamieson and Roberts, 2000). This swimming with increased frequency lasts for the duration of sensory input caused by the light dimming; if the light level returns to normal then swimming switches back to its normal frequency.

## 2 Model formulation

This sections begins with an explanation of the neurobiological details of sensory pathways and CPG neuron populations and their functional mechanisms in the Xenopus tadpole. Using these facts we formulate a model of interactive neuronal populations. Each population that has been included to the model corresponds to a set of neurons of some particular type in the real tadpole. The coupling between populations relates to experimentally measured neuronal connectivity.

The dynamics of the average level of activity in each population is described by the Wilson-Cowan equation (Wilson and Cowan, 1972). This equation is a simplification of real neurophysiological processes; however, this model reflects the most important details of dynamics at the population level. We derive equations for both sensory pathway and CPG networks and describe how the sensory pathway delivers information from external inputs to CPG populations to produce proper motor neuron activity.

### 2.1 Biological motivation for the model

In this section, we specify some neurobiological details of the tadpole nervous system which are important for the model formulation (Li et al., 2014). We consider two parts of the nervous system: sensory pathways (SP) and the central pattern generator (CPG).

#### 2.1.1 Sensory pathways

**Fig. 1:**
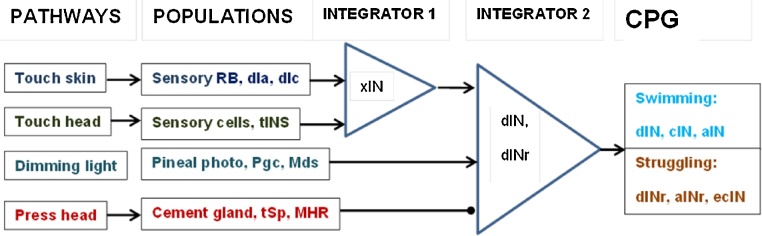
Schematic representation of the sensory pathways. Arrows represent excitatory synaptic connections, circles represent inhibitory connections.

We consider the following four sensory pathways (Fig. 1):

(1) Touch trunk skin (TS). This pathway includes Rohon-Beard (RB) cells, which are distributed along the rostro-caudal body dimension (about 100 neurons per side). In response to touching the trunk skin, several nearby RB neurons will each generate a single spike (Roberts et al., 2014). These spikes are propagated by the sensory pathway: dorso-lateral commissural and ascending neurons (dlc and dla, respectively). We assume that there is a population of interneurons (xINs) which receives signals from both dlc and dla neurons and tINs (see below). The population of xINs integrates incoming signals and delivers the excitation to dIN and dINr populations in the CPG.
(2) Touch head (TH). This pathway includes about 70 trigeminal sensory touch receptors (tSt) on each body side, which generate a single spike on touch. These sensor excite the neurons of the trigeminal nucleus (trigeminal inter-neurons; tINs) (Buhl et al., 2015). We assume that the tIN population excites the same integrating xIN populations as the trunk skin pathway (Fig. 1). The output from xIN populations builds up activity in the descending interneurons (dINs) of the CPG, potentially resulting in a decision to start swimming.
(3) Light dimming (LD). This pathway includes the photoreceptors of the pineal eye, which innervate pineal ganglion cells (pgc), and diencephalic/mesencephalic descending (D/Md) neurons on both body sides (Jamieson and Roberts, 1999). We assume that the output of the LD pathways converges onto dINs directly, bypassing the xIN populations. In response to light dimming, a tadpole starts swimming or, if already swimming, accelerates during the period of light dimming (Jamieson and Roberts, 2000).

Press head (PH) inhibitory pathway. Cement gland receptors (tSp) produce spikes on when pressure is applied to the head and innervate the inhibitory population of mid-hindbrain reticulospinal (MHR) neurons on both body sides (Perrins et al., 2002, Li et al., 2014). This inhibition converges to dINs and other CPG neurons (including motor neurons) to stop their activity.

Fig. 1 summarises the connections between populations in the sensory pathways. This connectivity is based on experimental evidence on cell level connectivity. Detailed reports of analysing this data as well as the computational modelling of inter-cellular connectivity are provided by publications: (Li et al., 2007, Roberts et al., 2014).

#### 2.1.2 Locomotor central pattern generator (CPG)

**Fig. 2:**
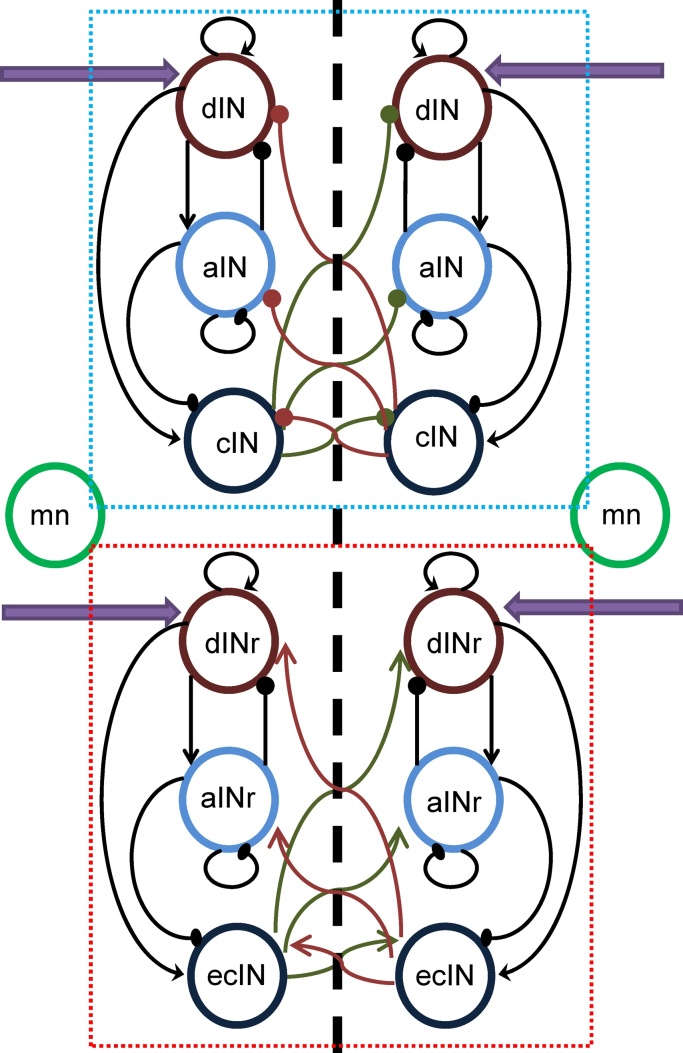
Schematic representation of CPG populations. The upper rectangular area (light blue dashed border) shows populations which are mostly active during swimming. The lower rectangular area (red dashed border) shows populations which are mostly active during struggling. Motor neurons are shown by green circles attached to both rectangular areas because the motor neurons receive connections from both swimming and struggling populations. (For interpretation of the references to colour in this figure legend, the reader is referred to the web version of this article.)

Here we consider the neural populations that generate locomotor behaviour. Fig. 2 shows the diagram of connections between CPG populations. We consider seven interacting populations on each body side. The upper rectangle (light blue dotted borders) contains the neuronal populations relevant to swimming (Roberts et al., 2014): excitatory descending interneurons (dINs), inhibitory ascending interneurons (aINs) and inhibitory commissural interneurons (cINs). The lower rectangle (red dotted borders) contains the populations related to struggling activity (Li et al., 2007): excitatory descending repetitive interneurons (dINrs), inhibitory ascending repetitive interneurons (aINrs) and excitatory commissural interneurons (eCINs). In addition, we consider a population of motor neurons, which receive multiple inputs from other CPG populations and are considered the output of the CPG.

Note that experiments suggest that there is one aIN population, containing some neurons that are more reliably spiking during swimming and some that are more active during struggling. To simplify the model we split the aINs into two populations: aINs in the swimming circuit and aINrs in the struggling circuit.

The populations of dIN and dINr neurons can be considered as decision-making populations: they integrate input from the sensory pathways and select a corresponding action from the repertoire. Swimming is characterised by the regular rhythmic activities of swimming populations with anti-phase oscillations between the equivalent populations on opposite body sides. The frequency of swimming is in the range 10–25 (Hz).

### 2.2 Model formulation: sensory pathways

1. We assume that the touch skin (TS) population includes sensory RB, dla and dlc neurons. We combine these populations into one model population per body side. The average activity in these combined populations on the left and right sides is denoted by X_1_(t) and X_2_(t) respectively.
2. Similarly, we assume that the TH population includes sensory tSt neurons and tINs, which are also combined together into one population per side. The average activity of this combined population on the left and right body sides is denoted by X_3_(t) and X_4_(t) respectively.
3. To model the light dimming (LD) pathway we combine photoreceptors, pgc and D/Md neurons into one population per side. The average activity of this combined population on the left and right side is denoted by X_5_(t) and X_6_(t) respectively.
4. We model the press head (PH) pathway as an inhibitory population, which includes the cement gland receptors and MHR neurons. The average activity of this combined population on the left and right side is denoted by X_7_(t) and X_8_(t) respectively.
5. To model the integration of sensory pathway activity we consider a population of xINs. The average activity of this population on the left and right body sides is denoted by X_9_(t) and X_10_(t) respectively.

To describe activity dynamics of ten neural populations corresponding to the four sensory pathways and one integrating population (per side), we use a mean field approach based on the Wilson-Cowan equations. The system of ordinary differential equations (ODEs) describing the sensory pathway activities is:

$$\begin{array}{c} \frac{dX_{j}}{dt}=-X_{j}+(k_{r}-X_{j})S_{r}(a_{j}\;X_{j}+P_{j}+\xi_{j}),\;\;\;r\in \left\{e,i\right\},\;\;\;j=1,...,8,\;\; \end{array}$$

$$\begin{array}{c} \frac{dX_{9}}{dt}=-X_{9}+(k_{e}-X_{9})S_{e}(a_{9}\;X_{9}+\lambda_{1}X_{1}+\lambda_{2}X_{3}+P_{9}+\xi_{9}) \end{array}$$

(1) $$\begin{array}{c} \frac{dX_{10}}{dt}=-X_{10}+(k_{e}-X_{10})S_{e}(a_{10}\;X_{10}+\lambda_{3}X_{2}+\lambda_{4}X_{4}+P_{10}+\xi_{10}) \end{array}$$

Here, *S_r_* (.) is the sigmoid function for either excitatory populations or inhibitory populations (indexed by *e* or *i* respectively), normalization parameters of excitatory and inhibitory populations *k_r_* = *S_r_*(+ ∞), *r* ∈ {*e*, *i*}. The formulas and parameter values for these functions as well as parameter values are given at Appendix A. All populations in (1) are excitatory except *X*_7_(*t*) and *X*_8_(*t*). Parameters *P_k_* , (*k* = 1, ..., 10) denote an external input to the corresponding population. Random Gaussian variables *ξ_k_* ∈ *N*(0, *σ*) , (*k* = 1, ..., 10) with mean zero and standard deviation *σ*, describe the noisy component of each sensory pathway. All other parameters (*a*_1_, ..., *a*_10_, *λ*_1_, ..., *λ*_4_) specify the connection strength either inside or between populations. All parameter values are given in Appendix A.

### 2.3 Model formulation: CPG

Fig. 2 shows a schematic representation of the CPG populations and their connections. The swimming and struggling networks are shown by rectangles with dotted blue and red borders respectively. Each black arrow shows a directed connection from one population to another, where a sharp arrow end denotes an excitatory connection and a round end denotes an inhibitory one. Commissural connections travel through the vertical dashed line to the opposite body side. The violet arrows to the dIN and dINr populations indicate multiple inputs (either excitatory or inhibitory) from the sensory pathways on each body side that deliver signals to control action selection. The output from the CPG network is a pair of motor neuron populations on the left and right body side, which receive connections from all CPG populations on the same side. The activity in the motor neuron populations is considered to represent the tadpole’s behaviour.

Thus, the CPG part of the model comprises three neuronal populations that are mostly active during swimming (dIN, aIN, cIN) on each side of the body. Their average activities we denoted by *Y*_1_(*t*), *Y*_2_(*t*), *Y*_3_(*t*) for the left side and *Y*_4_(*t*), *Y*_5_(*t*), *Y*_6_(*t*) for the right side. The other three CPG populations (dINr, aINr, ecIN) on each side of the body are mostly active during struggling. We denote their average activities by *Z*_1_(*t*), *Z*_2_(*t*), *Z*_3_(*t*) for the left side and *Z*_4_(*t*), *Z*_5_(*t*), *Z*_6_(*t*) for the right side. Motor neuron populations on the left and right body sides are denoted by *U*_1_(*t*), *U*_2_(*t*), respectively. These motor neurons receive inputs from other CPG populations and represent outputs of the CPG.

The equations for the swimming related populations on the left and right body sides are:

$$\begin{array}{c} \tau_{1}\frac{dY_{1}}{dt}=-Y_{1}+(k_{e}-Y_{1})S_{e}(w_{1}Y_{1}-w_{2}Y_{2}-\alpha_{1}Y_{6}+\nu_{1}X_{9}+\nu_{2}X_{5}-\nu_{3}X_{7}+Q_{1}) \end{array}$$

$$\begin{array}{c} \tau_{2}\frac{dY_{2}}{dt}=-Y_{2}+(k_{i}-Y_{2})S_{i}(w_{3}Y_{1}-w_{4}Y_{2}-\alpha_{2}Y_{6}+Q_{2}) \end{array}$$

$$\begin{array}{c} \tau_{3}\frac{dY_{3}}{dt}=-Y_{3}+(k_{i}-Y_{3})S_{i}(w_{5}Y_{1}-w_{6}Y_{2}-\alpha_{3}Y_{6}+Q_{3}) \end{array}$$

$$\begin{array}{c} \tau_{4}\frac{dY_{4}}{dt}=-Y_{4}+(k_{e}-Y_{4})S_{e}(w_{1}Y_{4}-w_{2}Y_{5}-\alpha_{1}Y_{3}+\nu_{1}X_{10}+\nu_{2}X_{6}-\nu_{3}X_{8}+Q_{4}) \end{array}$$

$$\begin{array}{c} \tau_{5}\frac{dY_{5}}{dt}=-Y_{5}+(k_{i}-Y_{5})S_{i}(w_{3}Y_{4}-w_{4}Y_{5}-\alpha_{2}Y_{3}+Q_{5}) \end{array}$$

(2a) $$\begin{array}{c} \tau_{6}\frac{dY_{6}}{dt}=-Y_{6}+(k_{i}-Y_{6})S_{i}(w_{5}Y_{4}-w_{6}Y_{5}-\alpha_{3}Y_{3}+Q_{6}) \end{array}$$

The equations for the struggling related populations on the left and right body sides are:

$$\begin{array}{c} \mu_{1}\frac{dZ_{1}}{dt}=-Z_{1}+(k_{e}-Z_{1})S_{e}(c_{1}Z_{1}-c_{2}Z_{2}+\beta_{1}Z_{6}+\delta_{1}sign\;(X_{9}-\Delta )sign\;(X_{10}-\Delta )+R_{1}) \end{array}$$

$$\begin{array}{c} \mu_{2}\frac{dZ_{2}}{dt}=-Z_{2}+(k_{i}-Z_{2})S_{i}(c_{3}Z_{1}-c_{4}Z_{2}+\beta_{2}Z_{6}-\delta_{2}sign\;(X_{9}-\Delta )sign\;(X_{10}-\Delta )+R_{2}) \end{array}$$

$$\begin{array}{c} \mu_{3}\frac{dZ_{3}}{dt}=-Z_{3}+(k_{e}-Z_{3})S_{e}(c_{5}Z_{1}-c_{6}Z_{2}+\beta_{3}Y_{6}+R_{3}) \end{array}$$

$$\begin{array}{c} \mu_{4}\frac{dZ_{4}}{dt}=-Z_{4}+(k_{e}-Z_{4})S_{e}(c_{1}Z_{4}-c_{2}Z_{5}+\beta_{1}Z_{3}+\delta_{1}sign\;(X_{9}-\Delta )sign\;(X_{10}-\Delta )+R_{4}) \end{array}$$

$$\begin{array}{c} \mu_{5}\frac{dZ_{5}}{dt}=-Z_{5}+(k_{i}-Z_{5})S_{i}(c_{3}Z_{4}-c_{4}Z_{5}+\beta_{2}Z_{3}-\delta_{2}sign\;(X_{9}-\Delta )sign\;(X_{10}-\Delta )+R_{5}) \end{array}$$

(2b) $$\begin{array}{c} \mu_{6}\frac{dZ_{6}}{dt}=-Z_{6}+(k_{e}-Z_{6})S_{e}(c_{5}Z_{4}-c_{6}Z_{5}+\beta_{3}Y_{3}+R_{6}) \end{array}$$

Finally, the equations for the motor neuron populations are:

$$\begin{array}{c} \kappa_{1}\frac{dU_{1}}{dt}=-U_{1}+(k_{e}-U_{1})S_{e}(\gamma_{1}Y_{1}-\gamma_{2}Y_{2}-\gamma_{3}Y_{6}+\gamma_{4}Z_{1}-\gamma_{5}Z_{2}+\gamma_{6}Z_{6}+\gamma_{7}U_{1}+M_{1}) \end{array}$$

$$\begin{array}{c} \kappa_{2}\frac{dU_{2}}{dt}=-U_{2}+(k_{e}-U_{2})S_{e}(\gamma_{1}Y_{4}-\gamma_{2}Y_{5}-\gamma_{3}Y_{3}+\gamma_{4}Z_{4}-\gamma_{5}Z_{5}+\gamma_{6}Z_{3}+\gamma_{7}U_{2}+M_{1}) \end{array}$$

Here, *S_e_*(.), *S_i_*(.) are the sigmoid functions for excitatory and inhibitory populations, respectively and their formulas and parameter values as well as parameter values of *k_e_* and *k_i_* are given at Appendix A. Parameters *w*_1_, ..., *w*_6_, α_1_, ..., α_3_, *v*_1_, ..., *v*_3_, *c*_1_, ..., *c*_6_, *β*_1_, ..., *β*_3_, *δ*_1_, *δ*_2_, *γ*_1_, ..., *γ*_7_ specify the connection strengths either inside or between populations. Parameters *μ*_1_, ..., *μ*_6_, *κ*_1_, *κ*_2_ define the characteristic time constants of population dynamics. To take into account the signal from LD pathway we assume that characteristic times *τ*_1_, ..., *τ*_3_ depend on the LD sensory signal *X*_5_ and parameters *τ*_4_, ..., *τ*_6_ depend on the LD sensory signal *X*_6_. Parameters *Q*_1_, ..., *Q*_6_, *R*_1_, ..., *R*_6_, *M*_1_, *M*_2_ define the external input to each population. Parameter Δ is the decision-making threshold of the integrating aINr and dINr populations. All parameter values are given in Appendix A.

Note that we do not take into account the spatial distribution of neurons along the body but instead consider a set of localised populations of neurons. In the model formulation, we use a minimal set of populations with a minimal number of connections between them representing the strongest synaptic pathways.

## 3 Dynamics of the sensory pathway model

In this section, we analyse the dynamics of the populations that process sensory input. Processing of input for a particular modality occurs in a single population, and the equation governing this population’s activity has the same form across all modalities. The same equation is also used to model the xIN populations, where input from the two touch pathways is integrated. The equation for a given population has the following form (extracted from Eq. (1)):

(3) $$\frac{dX}{dt}=-X+(k_{r}-X)S_{r}(a\;X+P+\xi )$$

Here, *X*(*t*) is the average activity of the sensory population at time *t*, *S_r_*(.) is the sigmoid function for either an excitatory population or inhibitory population (indexed by *e* or *i* respectively). The sigmoid function is characterised by two parameters: the threshold *θ*_*r*_ and the slope *b*_*r*_. Parameter *a* is the strength of self-coupling within the population, and parameter *P* is the amount of external input to the population. Normalization parameters *k*_*r*_ were defined above with formulas (1). The random Gaussian variable *ξ* ∈ *N*(0, *σ*), with mean zero and standard deviation *σ,* describes the noise component in each sensory pathway.

Thus, the 1D non-linear Eq. (3) includes four parameters. Depending on the parameter values, there are three possible dynamical regimes: (1) a stable state with constant low activity; (2) a stable state with constant high activity; (3) bi-stability, where stable low and high activities coexist for the same parameter values. In the bi-stable regime, the system demonstrates hysteresis under parameter variation, and a short-term external input moves the system form one stable state to another. We use this bi-stable regime to model the dynamics of sensory pathways and decision-making populations of integrating xINs.

**Fig. 3:**
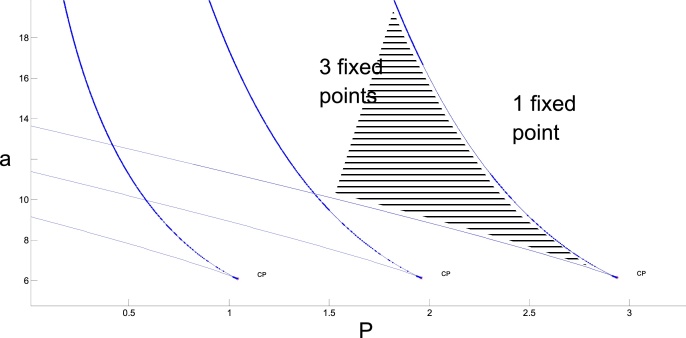
Superposition of 3 two-parametric bifurcation diagrams under variation of a third parameter (θ). The horizontal and vertical axes show parameter values of P and a (Eq. (3)). The rightmost curve shows the bifurcation diagram when *θ* = 5. Blue lines limiting the area filled by the horizontal line pattern correspond to saddle-node bifurcations (intersection of the line means that the saddle and the node fixed points merge and disappear). There are 3 fixed points in the patterned area (one saddle and two stable nodes) and outside of this area there is one stable node only. A similar explanation applies to the middle curve (this bifurcation diagram relates to *θ* = 4 and to the leftmost curve (*θ* = 3). Parameter *b* = 1.3 is fixed for all bifurcation diagrams. (For interpretation of the references to colour in this figure legend, the reader is referred to the web version of this article.)

To find parameter values corresponding to the bi-stable regime we plot 2D bifurcation diagrams under variation of parameters: *a*, *P* keeping the other two parameters fixed. The bifurcation diagrams are shown in Fig. 3.

Fig. 3 shows three bifurcation diagrams of co-dimension 2 corresponding to different values of *θ*. Each diagram has a cusp point (CP) where two saddle-node (SN) bifurcation lines merge. The area between these blue lines (shown by the horizontal line pattern in the rightmost bifurcation diagram) corresponds to a regime with three fixed points (two stable and one unstable). As the parameter values move towards the lines, the unstable (saddle) fixed point moves closer to one of the stable ones, and on the line the unstable and stable fixed points merge and disappear. Therefore, in the region outside of the lines there is a single stable fixed point, corresponding to either low or high constant activity depending on the parameter values.

To calculate the curve of saddle-node bifurcation on the co-dimension 2 bifurcation diagram, we used the software MATCONT (Dhooge et al., 2003) which is based on the idea of numerical continuation by parameters. In this particular case the saddle-node curve is described by two equations: Eq. (3) and another equation where the derivative of the right-hand side of Eq. (1) by the variable *X* equals to zero.

Generally the spiking activity of sensory neurons is noisy; we therefore include noise in the model of sensory populations (see formulas (1) and (3)): the external input is *P* + *ξ*. Here *P* is the constant part of input and *ξ* is a random Gaussian variable with zero mean and variance *σ*^2^. The value of the variance regulates the noise level; for example, the noise for pineal ganglion cells is substantially higher than the value of noise for other sensory pathways.

**Fig. 4:**
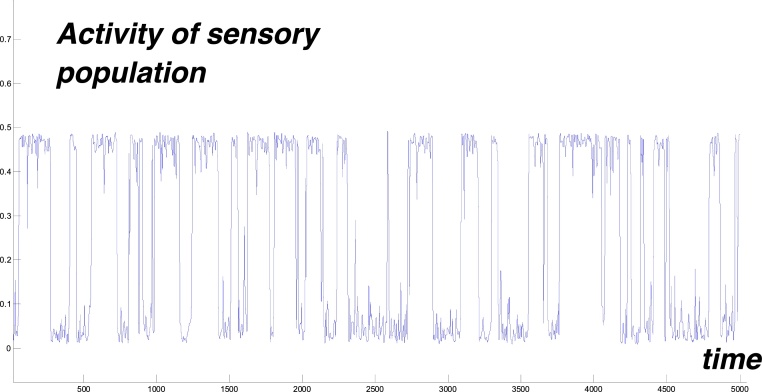
Noisy activity of sensory population vs time. There are multiple activity transitions between a low level (near zero) and a high level (near 0.5) Parameter values: *a* = 6, *P* = 1.2, *σ* = 0.2, *b* = 2, *θ* = 3.

If we consider parameter values inside the region with three fixed points then if the variance of noise is small, e.g. *σ* = 0.1, the system stays at either the low or high level fixed point. If the variance is higher, e.g. *σ* > 0.12 then the system will jump randomly between low and high levels of activity. To demonstrate this jumping we divide the interval of integration into a number of subintervals and on each subinterval with duration of 1 time unit, we randomly select a new value for the random Gaussian noise *ξ*. Fig. 4 the shows the resulting dynamics of population activity. This trajectory is typical for our model of sensory pathway activity.

If a sensory population receives external input (i.e. has a large value of $\bar{P}$) then the population spends most of the time in the high (active) state. The population returns back to the mostly low (rest) state when the input decreases. Both touch skin (TS) and touch head (TH) populations can initiate swimming or struggling, and their activities are integrated by the xIN population. If at least one of HS or TH populations is active then the xIN population becomes active and sends excitation to the CPG populations to initiate locomotor behaviour.

The LD populations receive a sensory signal from the pineal eye. We assume that both populations receive a similar excitation and might become temporally active. The activity of LD populations on both sides converges onto the CPG populations and, if tadpole is not moving, this short-term signal can initiate swimming. If the tadpole is already swimming then the signal from LD populations leads to swimming acceleration, i.e. the period of regular oscillations in the swimming network becomes smaller. The detailed neurobiological mechanism of swimming acceleration is not yet known. To model this phenomenon we assume that the characteristic times (*τ*_1_, …, *τ*_6_) of the swimming populations are modulated by the LD activity level.

The PC population receives an input signal from the press head and cement gland sensors and becomes active for the duration of the sensory signal. The activity of the PC population inhibits dINs and stops swimming activity.

It is known from experimental study of tadpole behaviour that swimming can start spontaneously, apparently without any sensory stimulation (Jamieson and Roberts, 2000). To model this we modify the equations governing the xIN populations. We consider the external input to xIN populations as a function of time, which slowly increases when the animal is in the resting state. The slope of this increase is randomly and uniformly selected from a range, such that the resulting delays to spontaneous swimming start match biological evidence. When the activity in the xIN populations reaches a threshold then swimming starts, even without input from the TS or TH pathways. After swimming starts, the activity of the xIN populations returns to a low level and stays constant during swimming or struggling.

The neurobiological mechanism by which swimming can spontaneously stop is unknown (some hypotheses see at Dale and Gilday, 1996, Zhang et al., 2015). To implement this behaviour we prescribe a maximum swimming duration. This duration is chosen randomly and uniformly from a suitable range. If the swimming is not disturbed by struggling then the swimming mode lasts until the end of the selected time interval.

## 4 Dynamics of CPG model

The CPG model can generate two main activity patterns: swimming and struggling. In a localised model the swimming pattern simply means the regular anti-phase oscillations of motor neurons (and other neuron types) on the left and right sides.

It is known from experiments that to initiate swimming a short touch of the skin on the trunk or head should be applied. This short touch provides a transient input (via the sensory pathway) to the dIN and dINr neurons on both sides of the body. Following the removal of sensory input, the CPG starts swimming and continues to work autonomously without additional signals from the sensory pathway. To model these dynamics we use a bi-stable regime, where a stable steady state (corresponding to low population activity/rest) coexists with a stable limit cycle (corresponding to anti-phase swimming oscillations) for the same parameter values. In this bi-stable regime, a short deviation of the system from the steady state leads to stable oscillatory activity.

To find appropriate parameter values for modelling swimming behaviour we used the results of bifurcation analysis of a Wilson-Cowan neural oscillator comprising two interacting populations (Borisyuk and Kirillov, 1992). A bi-stable region in parameter space has been found where a stable steady state and limit cycles (oscillations) co-exist, and we use parameter values from within this region.

In a localised model, struggling is characterised by anti-phase bursting activity: the bursts arise in anti-phase on the left and right sides of the body and exist for the duration of the input signal. We model struggling behaviour as a regime of envelope oscillations, corresponding to a torus in the multidimensional phase space of the dynamical system. This dynamical regime is characterised by two frequencies of oscillations: a high frequency of intra-burst spiking activity and a low frequency burst “envelope”. Motivation for using this type of dynamics comes from considering a population of more or less synchronously bursting neurons. The averaging of potentials of these bursting neurons will produce envelope oscillations with high and low frequencies. Example of such activity were considered in (Borisyuk, 2002, Fig. 2).

To find appropriate parameter values for modelling struggling behaviour we used the results of bifurcation analysis of two coupled Wilson-Cowan oscillators (Borisyuk et al., 1995). A regime of envelope oscillations was been found in this system and we use the corresponding parameter values for the model of struggling.

Now we report results of CPG model simulations with the selected parameter values. We assume that without external influences the CPG model is in the rest state and the activity of all populations is near zero. To initiate swimming in the CPG model we apply a short external input to the dIN population on one side, e.g. on the left side. This approach is rather artificial; a more natural way of initiating swimming is via a relevant sensory pathway. However, it is common in neurobiology to activate a neural activity by direct stimulation and in our computer experiments we adopt this approach.

**Fig. 5:**
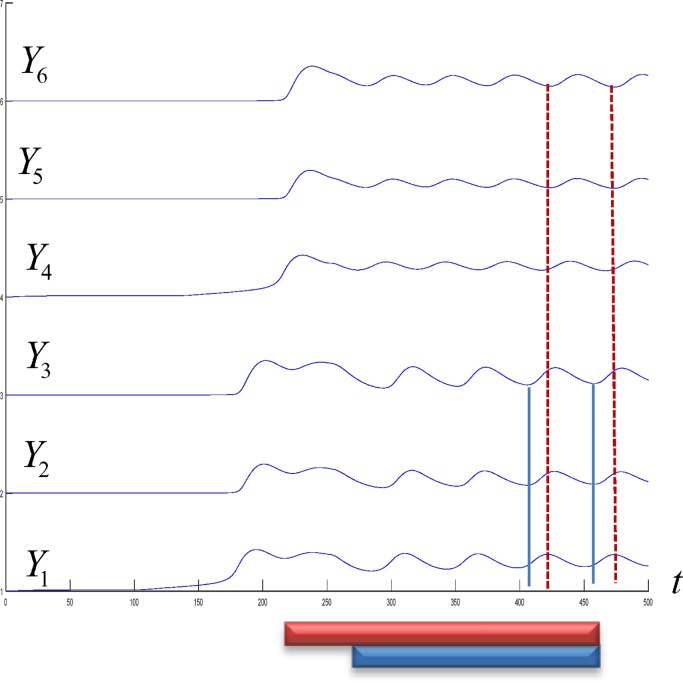
Swimming. Activities of dIN, aIN and cIN populations (from bottom to top) vs time during swimming. The lower three traces are from the left and upper three are from the right.

Experimental and modelling studies of swimming initiation following trunk skin stimulation provide detailed neuronal mechanisms of this process (Roberts et al., 2014). The coarse-grained population model provides a simplified description of swimming initiation. We assume that stimulation of the dIN population on one side combined with a delayed stimulation of the dIN population on the opposite body side will initiate swimming. Fig. 5 shows simulation results where swimming is initiated by a short stimulus on one side. An external input *Q*_1_ = 1.37 is applied to the dIN population on the left side from t = 100 until t = 250 ms (red horizontal bar in Fig. 5). To follow the experimental observations on appearance of activity on the opposite side, we apply an external input *Q*_4_ = 1.3 to the dIN population on the right side with delay of 30 ms, i.e. from t = 130 ms to t = 250 ms (blue horizontal bar in Fig. 5). To break symmetry, we use slightly different stimulation durations and amplitudes on the two sides. After the stimulation period (i.e. after 250 ms) the external input to both dIN populations returns back to the initial value *Q*_1_ = *Q*_4_ = 0.8. This constant non-zero value of external input corresponds to the depolarization of dIN neurons due to their relatively high background NMDA activation (Roberts et al., 2014).

**Fig. 6:**
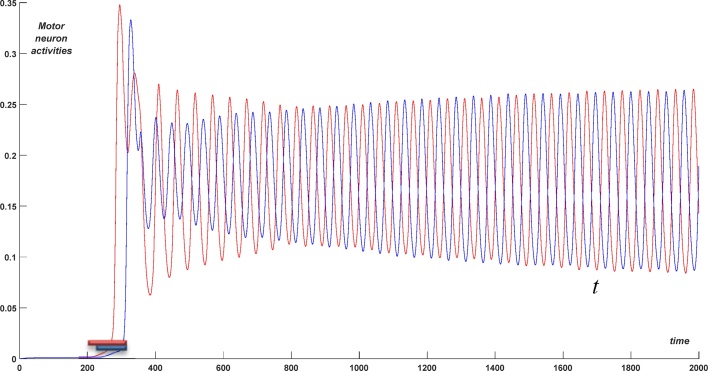
Swimming. Activity of motor neurons on the left (red) and right (blues) sides vs time. Red and blue short bars correspond to times of external input application to the left and right sides respectively. (For interpretation of the references to colour in this figure legend, the reader is referred to the web version of this article.)

**Fig. 7:**
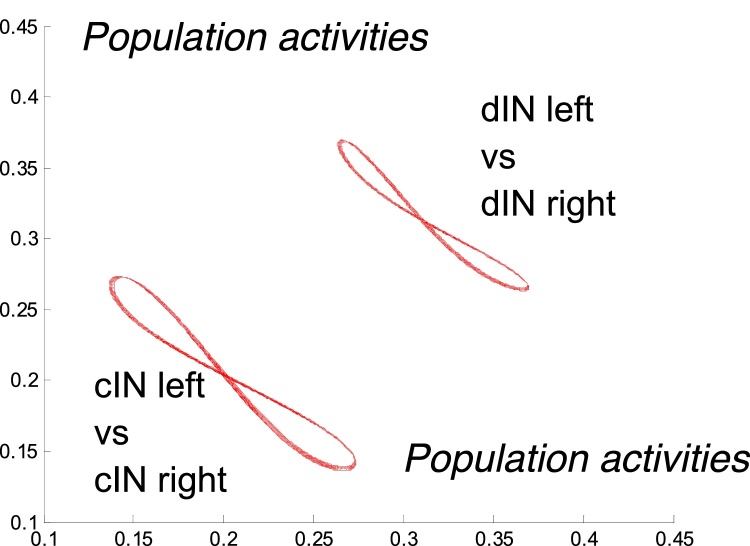
Swimming. Lower curve: Projection of the swimming limit cycle to the phase plane showing left and right cIN population activities. Upper curve: Projection of the limit cycle to the phase plane showing left and right dIN population activities. The shape of these curves is typical for anti-phase oscillations.

**Fig. 8:**
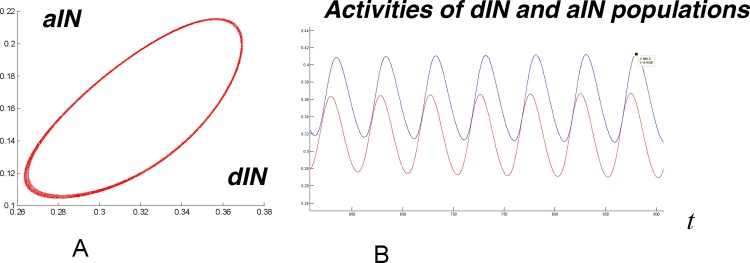
Swimming activities of dIN and aIN populations on the left side. A. Projection of the limit cycle to dIN (horizontal) and aIN (vertical) axes. B. Oscillatory activity of aIN (red) and dIN (blue) populations vs time. (For interpretation of the references to colour in this figure legend, the reader is referred to the web version of this article.)

Fig. 5 shows activity in the swimming populations (dINs, aINs, cINs) on the left (bottom traces) and right (top traces) in simulations with a short stimulus applied. Swimming activity starts on the side that is stimulated first, with the later stimulated side starting afterwards. The red and blue lines in Fig. 6 show activity in the motor neuron populations on the left and right sides respectively during the same simulation. It is clear that after a short transitional period with duration about 150 ms the system generates regular anti-phase oscillations with period *T *≈ 50 ms. Fig. 7 shows projections of two limit cycles to a 2D plane with like-wise populations on opposite sides. The larger limit cycle corresponds to antiphase oscillations in dIN populations on the left and right sides, while the smaller one shows anti-phase activity in cIN populations on the opposite sides. Fig. 8 shows the activities of dIN and aIN populations on the same side. Fig. 8A shows a projection of the limit cycle to the 2D plane of dIN (horizontal) and aIN (vertical) axes. The shape of the limit cycle is typical for in-phase oscillations with a small temporal shift. Fig. 8B shows oscillations of aINs (red) and dINs (blue). These two populations oscillate with a small phase shift of 10% of the period.

**Fig. 9:**
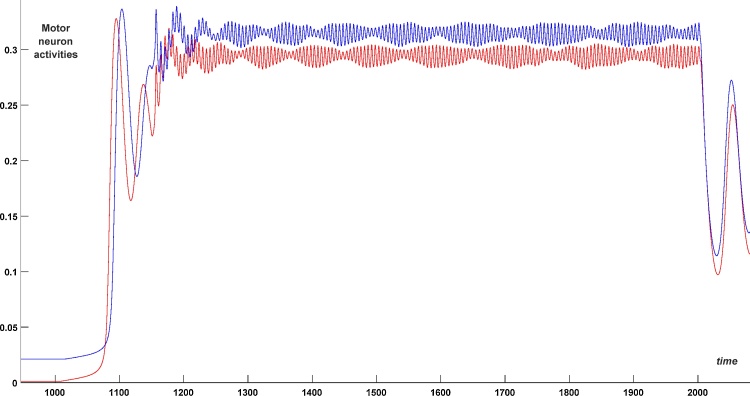
Struggling regime: Initiation of struggling from the rest position. Stimulation of dINr on both sides from 1000 to 2000 ms (1030–2000 for the right side). Motor neuron activity on the left (red) and right (blue) sides are shown vs time (blue curve was shifted up by 0.02 to avoid an overlap of graphs). (For interpretation of the references to colour in this figure legend, the reader is referred to the web version of this article.)

A simulation of struggling activity is shown in Fig. 9. Here, as before, we do not consider the sensory pathways but instead directly stimulate the dIN and dINr populations. To initiate struggling we apply a long-term stimulation for 1000 < *t* < 2000. Specifically, we stimulate the dIN population on the left side (red) for 1000 < *t* < 2000 and the dIN population on the right (blue) for 1030 < *t* < 2000 (see stimulation bars in Fig. 9). In addition, we stimulate the dINr populations on both sides for 1150 < *t* < 2000 (green stimulation bar). Because of this stimulation, modulated oscillations in the struggling network appear. The delay of 150 ms in stimulation of the dINr populations in comparison with the dIN populations distinguishes swimming and struggling regimes, since struggling starts only in response to prolonged stimulation of both body sides. Without such delay every swimming initiation will also initiate activity in the dINr populations. Therefore, if stimulation of dINs is less than 150 ms long then dINr neurons are not stimulated and the swimming regime will be generated. In case the dIN stimulation is longer than 150 ms, dINr stimulation is applied and struggling behaviour is produced.

Fig. 9 shows the activity in the motor neuron populations on the left (red) and right (blue) sides. Here the blue curve is shifted up by 0.07 units to avoid an overlap of graphs. The period of fast oscillations is about 5 ms and the period of the slow (envelope) oscillations is about 130 ms. These times are in line with experimental findings on struggling activity.

**Fig. 10:**
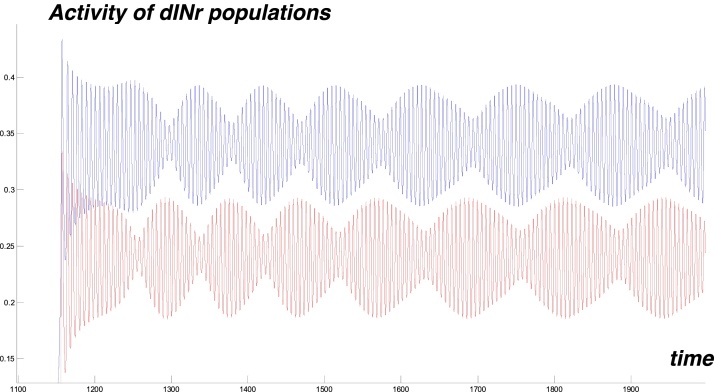
Struggling: Activities of dINr populations on left (red) and right (blue) body side vs time. Oscillations are shown with a vertical shift to avoid overlap. This figure demonstrates anti-phase oscillations at low frequency. (For interpretation of the references to colour in this figure legend, the reader is referred to the web version of this article.)

Fig. 10 shows activities of dINr populations on the left (red) and right (blue) sides during the same struggling simulation. The blue graph is shifted up to avoid overlap. These graphs show envelope oscillations with slow anti-phase oscillations between the two body sides. Populations of aINrs and eCINs have similar dynamics during struggling (not shown). When stimulation is stopped (at time 2000 ms) the system returns to generating swimming activity.

**Fig. 11:**
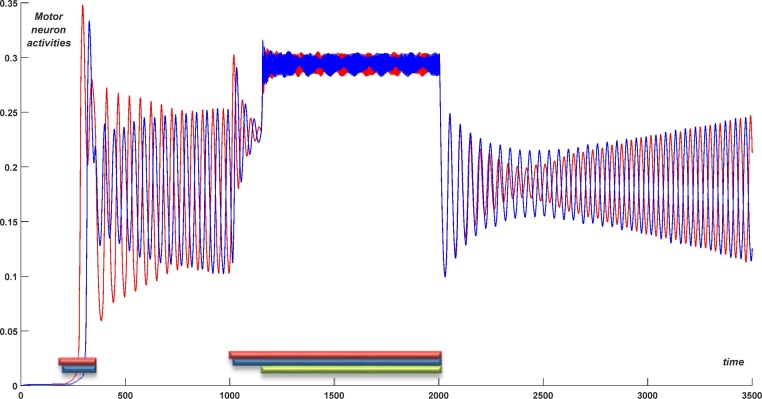
Struggling behaviour initiated from swimming. Stimulation was applied from 1000 to 2000 ms to excite dIN populations (red and blue bars) and dINr populations (green bar). Motor neuron population activity on the left and right sides is shown by red and blue lines, respectively. After the end of stimulation the CPG network returns to swimming. (For interpretation of the references to colour in this figure legend, the reader is referred to the web version of this article.)

Fig. 11 demonstrates that struggling activity can also appear during swimming. To initiate struggling we first initiate swimming (as described above) and then we apply a long-term stimulation to dINs and dINrs for time 1000 < *t* < 2000, also as described above. Fig. 11 shows slow anti-phase envelope oscillations of the motor neurons on the left (red) and right (blue) sides. As before, struggling only persists during stimulation, with activity returning to swimming after the stimulation ends. The end of struggling therefore always leads to swimming, regardless of the state before the stimulation (rest or swimming).

## 5 Modelling of tadpole behaviour

In this section, we present simulation of the complete model with sensory pathways and CPG populations. Before running the simulation, we prepare a scenario that describes a sequence of external influences to the model’s sensory inputs.

Here we describe one example scenario, which is summarized in Table 2. The scenario lasts from t = 0 to t = 7000 ms. We assume that the tadpole is initially in the steady state (at rest); therefore, at t = 0 all external inputs are initialized to zero. Event #1 is described as touching the skin on the left trunk during the time interval (200, 350) ms. Event #2 is light dimming in the time interval (1200, 1500) ms, etc. For each event in the scenario a relevant external input signal is generated. For example, for event #2, the input to TS sensory pathway changes for the duration of the event: the input to the TS population on the left side becomes 0.5 for 200 < *t* < 350 and the input to the right-side TS population becomes 0.1 for230 < *t* < 350. Event #2 causes swimming to start. Event #3 corresponds to light dimming, causing the input to the left and right LD populations to become non-zero for duration of event; as a result of this, swimming will be accelerated. However, it is possible that the swimming which was initiated by event #2 will stop spontaneously before the starting time of event #3. In this case the light dimming event will initiate swimming instead of accelerating it. Event #4 imitates a “predator attack”, which is represented as a prolonged stimulation to both body sides. As a result of event #4, struggling behaviour occurs. Event #5 means a signal to cement gland pressure receptors, meaning the HP sensory pathway becomes active, causing inhibition of swimming behaviour and return to the steady state.

**Table 2:** Scenario.

#	Initial time	Final time	Event
1	0	200	No events, tadpole is in the steady state
2	200	350	Short touch on the left side
3	1200	1500	Light dimming
4	2000	3000	Predator attack (struggling behaviour)
5	6500	6600	Pressure to head (stop signal)

**Fig. 12:**
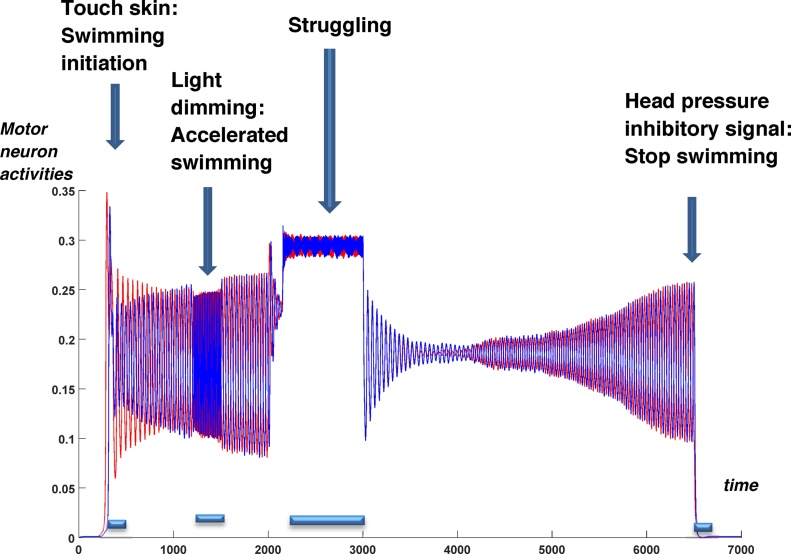
Activity of motor neuron populations on the left (red) and right (blue) body sides. These activities show a sequence of behaviours arising according the scenario events. (For interpretation of the references to colour in this figure legend, the reader is referred to the web version of this article.)

Fig. 12 shows activity in the motor neuron populations vs time on the left (red) and right (blue) sides corresponding to the events in Table 1. Events are shown by vertical arrows. The duration of each event is shown by the horizontal bar under the time axis. These events represent a sequence of environmental signals to the tadpole’s sensors, which modulate the dynamics of the corresponding sensory pathways. As a result of these influences from the sensory pathways, the CPG populations generate the relevant population dynamics. Motor neuron populations represent the output of the CPG and their activities demonstrate how the tadpole behaviour reflects the incoming environmental signals (events) which are specified in the scenario. The model dynamics are modulated by these signals to make decisions on appropriate locomotor actions and behaviour in response to environmental signals.

**Table 1:** summarises description of four sensory pathways. For each pathway the names of populations in that pathway are given, along with the names of corresponding dynamic variables in the model. These variables describe the dynamics of pathway populations.

Pathway	Combined population	Model activity variable on left and right body side respectively
Touch skin (TS)	Sensory Rohon-Beard (RB) cells; dorso-lateral commissural (dlc) and ascending (dla) neurons	*X*_1_(*t*) and *X*_2_(*t*)
Touch head (TH)	trigeminal sensory touch receptors (tSt); trigeminal nucleus inter-neurons (tIN)	*X*_3_(*t*) and *X*_4_(*t*)
Light dimming (LD)	Photo receptors, pineal ganglion cells (pgc), Diencephalic/Mesencephalic descending (D/Md) neurons	*X*_5_(*t*) and *X*_6_(*t*)
Press head (PH)	Cement gland receptors (tSp); inhibitory mid-hindbrain reticulospinal neurons (MHR)	*X*_7_(*t*) and *X*_8_(*t*)

## 6 Discussion

In this paper, we have presented a computational model that is able to produce a set of behaviours mimicking the locomotor activities in a young tadpole (Roberts et al., 2010). These model behaviours arise in response to incoming signals from the “environment” according to a scenario consisting of a sequence of events. Thus, a complete meso-scale model of the tadpole nervous system has been developed. This model is able to process multiple sensory inputs and integrate them to make decisions about what locomotor response should be generated. The model is deeply rooted to data from many neurobiological experiments. For example, the mechanism of swimming initiation in response to skin touch has been studied and modelled at the level of spiking activity, and the neuronal populations participating in this process have been identified (Roberts et al., 2014, Buhl et al., 2015).

The model contains four sensory pathways as well as integrator and CPG populations. Although many experimental facts about the neurons in the sensory pathways and their electrophysiological properties are available, there are still many open questions and uncertainties, which require further investigation. Therefore, although we cannot derive a detailed model of spiking neurons for sensory pathways nevertheless, we use another approach that is based on a population model of Wilson-Cowan type. For consistency, the same approach has been used for modelling CPG activity.

Population level (mesoscopic-scale) models (also known as neural-mass models) are widely used in computational neuroscience. Several different approaches have been developed, but all consider neuronal activity in entire neuronal populations (Wilson and Cowan, 1972, Jansen and Rit, 1995, Marten et al., 2009, Liley et al., 2010). In this approach, interacting populations influence each other by excitatory or inhibitory connections, which is usually represented by a sigmoid function (Marreiros et al., 2008). If the distribution of population’s activity in space is considered then a generalization of neural-mass model known as a neural-field (mean-field) approach is applicable (Bressloff, 2012, Hlinka and Coombes, 2012). A natural extension to our model would be to consider spatial information, to attempt to reproduce the head-to-tail propagation of swimming activity that is seen in experiments, for example.

Decision making in our model is based on a slow increase of population activity toward a threshold. The activity of sensory populations is noisy and there is therefore variability in time of first threshold passage in the integrating population (Wang, 2002). This approach is in line with theoretical ideas on decision making in neuronal circuits (Kristan, 2008, Gold and Shalden, 2007, Larsen and Bogacz, 2010, Marshall et al., 2012, Onslow et al., 2014).

Swimming in the model is based on a bi-stable regime. A short excitatory influence moves the system from a stable steady state to stable oscillations. Similarly, a short inhibition moves the system back to the resting steady state. Struggling modelling is characterised by bursting activities of participating neurons, therefore, it is appropriate to describe population activity during struggling by envelope oscillations with fast and slow frequencies.

Simulation of the model shows complex behaviour even in the case of a pre-defined scenario of input events. The reason is that the model includes spontaneous starting and stopping of swimming as well as random noise in the sensory populations. Therefore, repeated model simulations with the same parameter values according to the same scenario lead to different dynamics and behaviours. This makes the model behaviour less predictable and more similar to real tadpole movements in natural environments. Let us assume two robotic tadpoles each controlled by the population model. Even if both robots are initiated at almost the same time and receive the same inputs, their behaviours might become significantly different after a short period of time. This fact means that the system is characterised by a complex and unpredictable behaviour, like a real tadpole.

The population level model demonstrates a good correspondence between input events and behaviour. A further model development will include much more biologically realistic modelling of spiking neurons and reflect recent experimental findings regarding the functioning of sensory pathways and integrators (Buhl et al., 2015, Koutsikou et al., 2016). In addition, we would like to mention a possible implementation of the population model as a swimming robotic tadpole. An advantage of the population model over a detailed spiking neuron model is that the population model is much more suitable for robotic implementation since it requires much less computational power, making real-time simulation possible.

## Figures and Tables

**Table A1:** Model parameters and their values.

P	Val	P	Val	P	Val	P	Val	Par	Val	P	Val	P	Val	P	Val
$a_{1}$	6	$P_{1}$	0.8	$\lambda_{1}$	1.8	$Q_{1}$	0.8	$\nu_{2}$	0	$R_{1}$	0.8	$\tau_{1}$	1	$\mu_{5}\;$	1
$a_{2}\;$	6	$P_{2}\;$	0.8	$\lambda_{2}$	1.8	$Q_{2}\;$	0	$\nu_{3}$	2	$R_{2}\;$	0.8	$\tau_{2}\;$	1	$\mu_{6}\;$	1
$a_{3}\;$	6	$P_{3}\;$	0.8	$\lambda_{3}$	1.8	$Q_{3}\;$	0	$c_{1}\;$	16	$R_{3}\;$	0.8	$a_{4}\;$	1	$\kappa_{1}$	3
$a_{4}\;$	6	$P_{4}\;$	0.8	$\lambda_{4}$	1.8	$Q_{4}\;$	0.8	$c_{2}\;$	12	$R_{4}\;$	0.8	$a_{4}\;$	1	$\kappa_{2}\;$	3
$a_{5}\;$	6	$P_{5}\;$	0.9	$w_{1}\;$	16	$Q_{5}\;$	0	$c_{3}\;$	15	$R_{5}\;$	0.8	$\tau_{5}\;$	1	$M_{1}\;$	0.1
$a_{6}\;$	6	$P_{6}\;$	0.8	$w_{2}\;$	12	$Q_{6}\;$	0	$c_{4}\;$	3	$R_{6}\;$	0.8	$\tau_{6}\;$	1	$M_{2}\;$	0.1
$a_{7}\;$	6	$P_{7}\;$	0.9	$w_{3}\;$	10.9	$\alpha_{1}$	0.1	$c_{5}\;$	16	$\delta_{1}$	1	$\mu_{1}$	1	$b_{e}\;$	1.3
$a_{8}\;$	6	$P_{8}\;$	0.8	$w_{4}\;$	3	$\alpha_{2}\;$	0.2	$c_{6}\;$	3	$\delta_{2}$	1	$\mu_{2}\;$	1	$b_{i}\;$	2
$a_{9}\;$	6	$P_{9}\;$	0.8	$w_{5}\;$	11	$\alpha_{3}\;$	0.1	$\beta_{1}$	0.9	Δ	5	$\mu_{3}\;$	1	$\theta_{e}\;$	4
$a_{10}\;$	6	$P_{10}\;$	0.8	$w_{6}\;$	2	$\nu_{1}$	0	$\beta_{2}$	0	$v_{3}$	2	$\mu_{4}\;$	1	$\theta_{i}\;$	3.7
$\gamma_{1}$	10	$\gamma_{2}$	0.5	$\gamma_{3}$	0.5	$\gamma_{4}$	3	$\beta_{3}$	0.1	$\gamma_{5}$	2	$\gamma_{6}$	1	$\gamma_{7}$	0.1

## Appendix A

Sigmoid functions of excitatory and inhibitory populations (indexed by *e* or *i* respectively) are: $S_{r}(x)=\frac{1}{1+e^{-b_{r}(x-\theta_{r})}}-\frac{1}{1+e^{b_{r}\theta_{r}}}$, *r* ∈ {*e*, *i*}. The normalization parameters of excitatory and inhibitory populations (indexed by *e* or *i* respectively) are: *k*_*r*_ = *S*_*r*_(+ ∞), *r* ∈ {*e*, *i*}.

Random Gaussian variables *ξ*_*k*_ ∈ *N*(0, *σ*) , (*k* = 1, ..., 10) with the mean equal zero and the standard deviation *σ* = 1 describe the noise components of sensory pathways.

All other parameters and their value are given in Table A1. To break symmetries we randomise all parameters by adding white noise with variance equal to 0.01.

Parameter value choice is an important part of model development. We use three main approaches to this. (1) Parameter value is motivated by experimental measurements. This approach has been mostly used for parameter values of equations describing the activity dynamics of sensory pathways and references to experimental papers are given in Section 2.1. (2) Parameter value is selected according to theoretical (bifurcation theory) studies. We mostly use this approach to select parameter values for the swimming and struggling networks. Modelling of swimming is based on a bistable regime where a stable fixed point co-exists with a stable limit cycle. In (Borisyuk and Kirillov, 1993) we found this regime in a system describing a Wilson-Cowan oscillator (interacting excitatory and inhibitory populations) and starting from these basic parameter values we select values in the swimming network to provide the bistable regime. In a similar way, for modelling the struggling behaviour we use envelope oscillations with a high frequency related to spiking inside bursts (synchronous bursting on one body side) and a low frequency corresponding to the slow escaping movement. Of course, high frequency oscillations should be antiphase between the two body sides. In (Borisyuk et al., 2003) we found this regime of envelope oscillations in a system of two coupled Wilson-Cowan oscillators and we modify these basic parameter values to get anti-phase envelope oscillations in the struggling network. (3) Parameter value is selected as a result of multiple computational model simulations. This approach has been used in a few cases where neither inspiration from experiments nor from theory were available.
